# High level of inter-species transmission of simian foamy virus from non-human primates to humans in Gabon, central Africa

**DOI:** 10.1186/1742-4690-8-S1-A229

**Published:** 2011-06-06

**Authors:** Augustin Mouinga-Ondémé, Mélanie Caron, Antoine Gessain, Mirdad Kazanji

**Affiliations:** 1 Unité de Rétrovirologie, CIRMF, Franceville, Gabon; 2 EPVO unit Institut Pasteur, Paris, France; 3 Unité de Rétrovirologie, CIRMF, Franceville, Gabon and Institut Pasteur de Bangui, Central Africain Republic

## Background

Each of the pathogenic human retroviruses (HIV-1/2 and HTLV-1) has a non-human primate (NHP) counterpart, and the presence of these retroviruses in humans results from interspecies transmission. The passage of another simian retrovirus, simian foamy virus (SFV), from NHPs to humans has been reported. Here, we evaluated the natural history of SFV in a free-ranging colony of mandrills (CIRMF primate center) and in mandrills living in natura in Gabon (central Africa). We also determined the SFV prevalence in a series of 497 NHP living in different parts of Gabon. Lastly, we investigated the possible transmission of SFVs to humans.

## Results

Seropositivity for SFV was Western blot positive in 83% (70/ 84) of captive and 60% (9/15) of wild-caught mandrills. Integrase gene analysis demonstrated the existence of two different, geographically restricted, MndFV strains. Among the NHP, 10.5% (31/286) of the plasma/sera were SFV seropositive. Integrase gene was characterized in 38 samples with novel SFVs in several species of Cercopithecus. Among the 78 persons, mostly hunters bitten by NHP, 19 were SFV seropositive with 15 being PCR confirmed. Mandrills and gorilla SFV were found in the infected humans. Furthermore, SFV was detected in two personnel of the primate center.

## Conclusion

We demonstrate the presence of 2 different geographically restricted strains of mandrill FV. We show the existence of several new FV strains, species-related, in different Cercopithecus. Lastly, our results indicate a high interspecies transmission of SFVs to hunters through mainly gorilla bites, leading to a chronic infection in humans.

